# The Prevalence of Chronic Interosseous Membrane Lesions Following Mason II and III Radial Head Fractures in Complex Elbow Instability—A Retrospective Observational Cohort Study

**DOI:** 10.3390/healthcare12181875

**Published:** 2024-09-19

**Authors:** Giuseppe Giannicola, Luca Di Sante, Giulia Corsi, Carmine Zoccali, Sebastien Prigent, Gianluca Cinotti, Pasquale Sessa

**Affiliations:** 1 Department of Anatomical, Histological, Forensic Medicine and Orthopedics Sciences, “Sapienza” University of Rome, 00185 Rome, Italy; giuseppe.giannicola@uniroma1.it (G.G.); luca.disante@uniroma1.it (L.D.S.); carmine.zoccali@uniroma1.it (C.Z.); sebastien.prigent@uniroma1.it (S.P.); gianluca.cinotti@uniroma1.it (G.C.); 2 Department of Orthopedics and Traumatology, Cristo Re Hospital, 00167 Rome, Italy; giulia.corsi@gmail.com; 3 Department of Orthopedics and Traumatology, San Camillo-Forlanini Hospital, Circonvallazione Gianicolense 87, 00100 Rome, Italy

**Keywords:** central band, chronic injury, complex elbow instability, interosseous membrane lesion, IOM, muscular hernia sign, radial head fracture

## Abstract

Purpose: The primary aim of the present study was to assess the prevalence of chronic lesions of the central band of the interosseous membrane (cbIOM) in complex elbow instability (CEI) in a consecutive series of patients who had previously undergone surgical treatment for Mason II and III radial head (RH) fractures. The secondary aim was to define its clinical significance. Methods: We performed a retrospective study on a prospective database. Our study population comprised 93 patients affected by CEI with type II or III RH fractures according to Mason’s classification who were analyzed in the chronic setting. All patients were treated according to the current therapeutic algorithms. At the last follow-up, the “muscular hernia sign” was investigated by means of a bilateral ultrasonographic examination to assess any chronic cbIOM lesions; the Mayo Elbow Performance Score (MEPS) was used to evaluate the clinical significance of these lesions. Results: All 93 patients were assessed after a mean time of 7.3 years (range: 2–12). No positive “hernia signs” were found, while five patients (5.4%) displayed an increased laxity of the cbIOM when compared with the contralateral side despite a negative “hernia sign”. The clinical outcome in all five patients was excellent with a mean MEPS of 96 (range, 90–100). Conclusions: Chronic cbIOM lesions are very rare in CEI with RH fractures. No patients in this large sample displayed a cbIOM complete lesion; in cases with increased laxity, satisfactory mid-term clinical results were observed. Considering that previous studies reported (1) a high prevalence of cbIOM lesions in patients with Mason II and III RH fractures and (2) the current expert opinion about the scarce healing potential of the cbIOM, this study also suggests that the IOM may heal better than previously believed when RH fractures are treated appropriately in the acute setting.

## 1. Introduction

Forearm stability relies on three main anatomical structures, which are the proximal radio-ulnar joint, the interosseous membrane (IOM), and the distal radio-ulnar joint [1,2,3,4,5]. The central band of the IOM (cbIOM) is credited to be the most important part of this structure on account of the longitudinal and transversal forearm stability it provides, as well as for the transfer of loads from the wrist to the elbow joint [1,2,6]. When a multifragmentary fracture of the radial head occurs, the cbIOM remains the only structure that can ensure the proper longitudinal and transverse relationship between the radius and ulna; if a radial head (RH) resection is performed in the presence of a disruption of the cbIOM, severe longitudinal forearm instability occurs, leading to an Essex-Lopresti lesion [7,8,9,10,11]. 

The importance of the cbIOM was recently highlighted by clinical observations of an association between undiagnosed cbIOM lesions and chronic lateral elbow pain in patients affected by acute complex elbow instability (CEI) whose radial head fractures were adequately treated by means of prosthetic replacement or reconstructive procedures [12,13]. A modified load transfer pattern from the wrist to the elbow due to chronic IOM insufficiency, combined with radio-humeral joint overloading and degeneration, was hypothesized to be the cause of lateral elbow pain; consequently, several reconstructive procedures for the cbIOM have recently been proposed to treat such cases [8,9,14,15]. However, no studies have thoroughly assessed the prevalence of chronic cbIOM lesions in a consecutive series of patients after appropriate treatment of RH fractures in acute CEI to better understand the clinical significance of these lesions.

Moreover, another relevant issue about the cbIOM is represented by its healing potential after injuries. Since no studies are available on this topic, the current expert opinion is that the cbIOM does not heal after a complete rupture, leading to an important alteration in the radio-ulnar load-sharing mechanism of the forearm [14,16]. For this reason, some authors have started to perform the reconstruction of cbIOM in the acute setting [17]. Considering that recent MRI studies on radial head injuries have reported a high prevalence of IOM lesions in the acute setting [18,19], a high number of patients with chronic cbIOM insufficiency should be expected after complex elbow instability (CEI) associated with RH fractures. A better understanding of this troublesome issue has relevant clinical implications since the reconstruction of the cbIOM in the acute setting may represent an overtreatment if a better healing potential was demonstrated.

Starting from these observations, the present study aimed (1) to assess the prevalence of a chronic cbIOM lesion in a large series of patients affected by CEI and treated in the acute phase according to the current therapeutic algorithms and (2) to define the clinical significance of the cbIOM lesions observed. On the basis of the current knowledge on this topic, the null hypothesis of the present study was that a high prevalence of symptomatic chronic cbIOM lesions would emerge.

## 2. Materials and Methods

**Figure 1:**
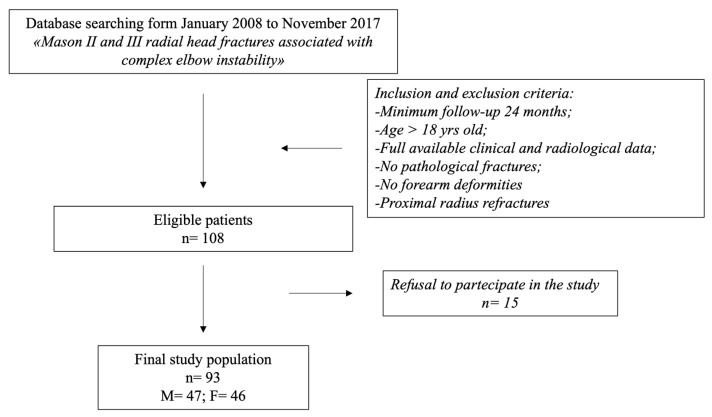
A flowchart of the patient inclusion process.

This is a retrospective study on a prospective database of patients affected by acute CEI and surgically treated by a single expert elbow surgeon (GG) between January 2008 and November 2017. The inclusion criteria were patients with acute complex elbow instability with type II or III radial head fractures according to Mason’s classification [20]; a minimum follow-up of 24 months; patients aged > 18 years; and full available clinical and radiological data. The exclusion criteria were proximal radius refractures; pathological fractures; and forearm deformities. Overall, 108 consecutive patients were identified; 55 patients presented a terrible triad injury (i.e., elbow dislocation, coronoid, and radial head fracture), 33 patients presented a Monteggia-like fracture-dislocation (i.e., fracture of the proximal ulna and radial head fracture-dislocation), and 21 patients presented an elbow dislocation with radial head fracture (Mason type II or III), and all were treated in the acute setting according to the most recent therapeutic algorithms [21,22,23,24]. All 108 patients were contacted by phone after a mean time of 7.3 years (range: 2–12) following the surgical procedure and invited to undergo a clinical and ultrasonographic (US) examination. A total of 93 of the 108 patients accepted; the final study group consisted of 47 males and 46 females. The patient inclusion flowchart is shown in Figure 1. The injury pattern consisted of 51 terrible triad injuries, 22 Monteggia-like lesions, and 20 elbow dislocations with radial head fractures. Overall, there were 67 Mason type III and 26 Mason type II radial head fractures. A bilateral US examination of the forearm was performed by a single examiner (LDS) who is skilled in musculoskeletal ultrasonography. A Samsung HS 60 ultrasound system with a 10 MHz linear probe was used. Written informed consent was obtained from all participants of this study. The statistical analysis was performed by one of the authors (PS). The STROBE reporting checklist for observational studies was used for the present study.

### Ultrasonographic Examination

**Figure 2:**
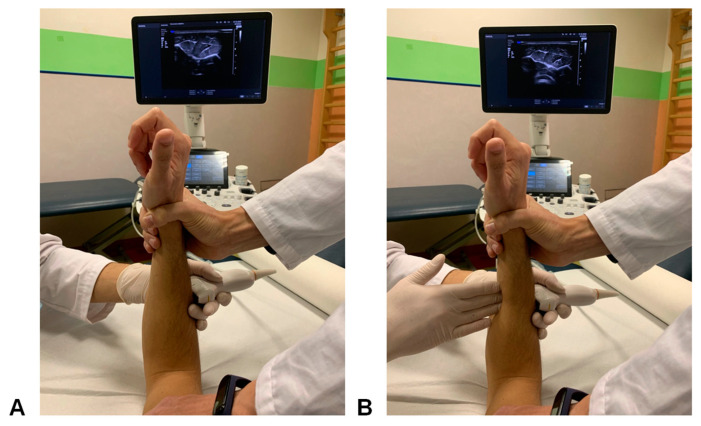
The “hernia sign test” performed with the forearm in neutral rotation and the elbow flexed at 90°; the US probe is placed dorsally on the middle third of the forearm (**A**) and a compression is applied by the examiner on the ventral side of the forearm to assess whether a flexor mass protrusion in the extensor compartment is present (**B**). A flexor mass protrusion in the dorsal compartment of the forearm indicates a cbIOM insufficiency and a “positive hernia sign”.

The exam was performed with the patient sitting and the upper arm lying on the examination table, as described by Soubeyrand et al. [25]. The forearm was in a neutral rotation, and the elbow was flexed at 90° (Figure 2). A two-step examination was performed. In the first part of the exam, a static morphological evaluation of the interosseous membrane was performed, including a comparison between the injured and the non-injured side. The linear probe was positioned perpendicular to the forearm longitudinal axis on the dorsal part, and a proximal-to-distal examination was performed to assess the characteristics of the cbIOM. Differences in morphology, thickness, continuity, and echoic aspect between the two sides were assessed to identify any sign of a previous lesion as described by previous studies [26,27,28,29].

In the second part of the examination, a comparative dynamic assessment of the cbIOM tension was performed by means of the “hernia sign” test, as described by Soubeyrand et al. [25] in 2006. A 100% reliability of the “muscular hernia sign” was reported in detecting surgically produced cbIOM lesions in fresh-frozen forearms in an experimental setting [28]. The linear probe was positioned on the dorsal aspect of the middle third of the forearm, and digital compression was exerted on the volar aspect of the forearm to assess whether a flexor muscle mass protrusion into the extensor compartment was present; if so, herniation of the flexor muscles was considered as a complete cbIOM lesion. If the “hernia sign” test was instead negative, any differences in cbIOM laxity between the two sides were recorded as “symmetrical laxity”, “increased laxity”, and “decreased laxity”. 

In patients with any kind of alteration in the cbIOM at the ultrasonographic static and dynamic assessment (i.e., signs of membrane discontinuity or increased laxity), a clinical and radiographic examination was performed by using the Mayo Elbow Performance Score and Index (MEPS and MEPI, respectively); additionally, anteroposterior and lateral X-ray examinations of the forearm were included to analyze the radio-capitellar joint and the radio-ulnar joints.

## 3. Results

**Figure 3:**
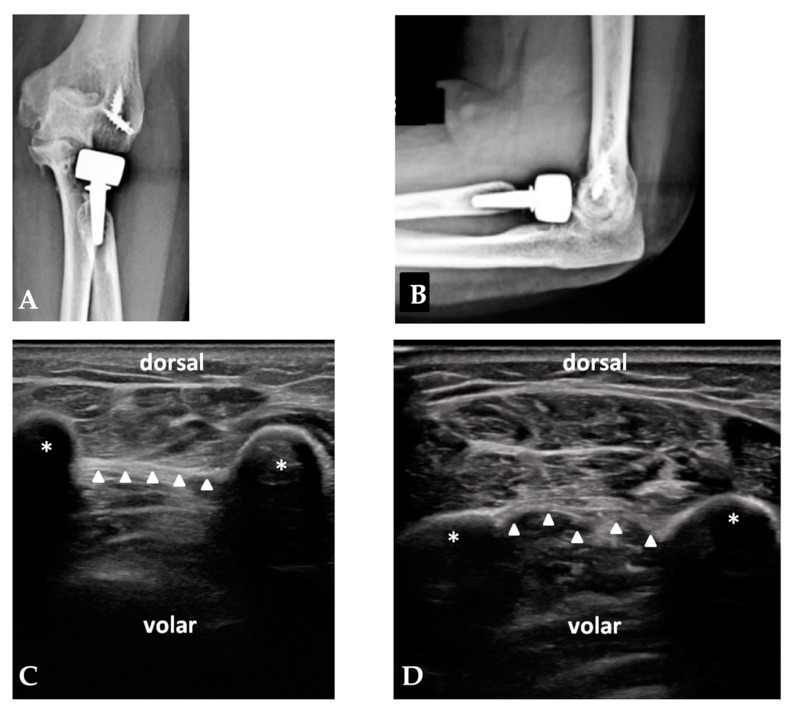
Terrible triad injury of elbow in 57-year-old female patient, left side. Post-operative AP and lateral X rays (**A**,**B**) at last follow-up: bipolar radial head arthroplasty was implanted and transosseous suture was performed to fix coronoid fracture; lateral collateral ligament and posterolateral capsule were repaired with two 5 mm suture anchors. At last follow-up, bilateral US examination of cbIOM was performed. (**C**) shows dynamic examination of non-injured side, while (**D**) shows increased laxity on affected side. White asterisks represent forearm bones. White arrowheads represent interosseous membrane.

**Figure 4:**
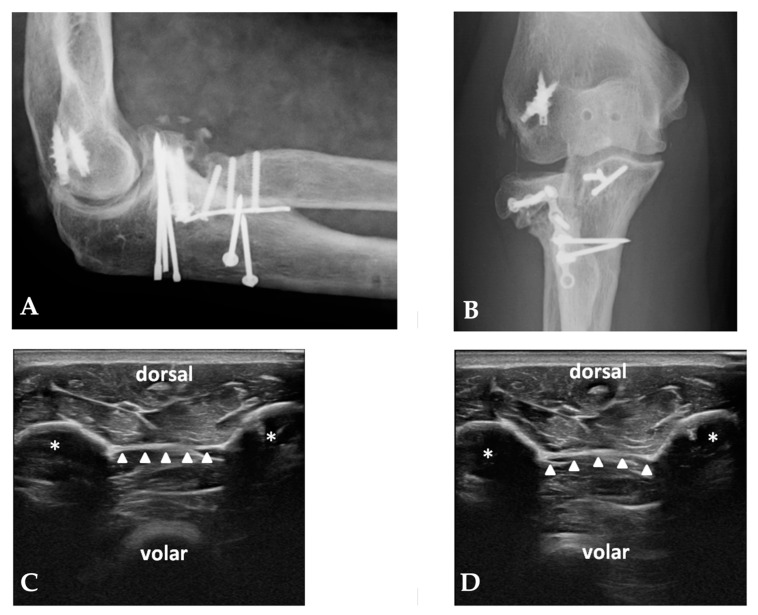
A Monteggia-like lesion in a 60-year-old female patient. The last follow-up of the patient after reintervention for contracture release and posterior ulnar plate removal. The X-rays show (**A**,**B**) an asymptomatic non-union of the radial head and radial plate breakage. At the last follow-up, a bilateral US examination of the cbIOM was performed. (**C**) shows a dynamic examination of the non-injured side, while (**D**) shows a slight increase in laxity on the affected side. The white asterisks represent the forearm bones. The white arrowheads point to the interosseous membrane.

The mean follow-up was 7.3 years (range: 2–12 years). The mean age of the patients was 57.5 years (range: 23–76). None of the 93 patients displayed a positive “hernia sign”, while five patients (5.4%) displayed an increased laxity of the cbIOM when compared with the non-injured side despite a negative “hernia sign” test. In particular, among these five patients, three patients showed a Monteggia-like fracture injury pattern, one patient showed a terrible triad pattern, and one patient showed the injury pattern of RH fracture associated with elbow dislocation. In the remaining 88 patients, no side differences were assessed regarding the morphology, thickness, echoic aspect, and continuity of the cbIOM. The general characteristics, the diagnosis, and the clinical and radiographic results of the five patients with an increased hernia sign are shown in Table 1 and in Figure 3 and Figure 4.

## 4. Discussion

The aim of the present study was to assess the prevalence and clinical significance of chronic cbIOM lesions in a sample of patients affected by Mason II and III RH fractures in CEI and surgically treated according to the current algorithms [21,22,23,24]. The results observed suggest that chronic cbIOM lesions are exceedingly rare considering that no cases were observed in this large sample, questioning if the prevalence of acute cbIOM lesions is lower than previously described or its potential healing is higher than previously supposed. In both cases, this study shows that the IOM insufficiency in CEI is a rare clinical condition in the chronic setting when fracture-dislocations are properly addressed in the acute phase.

Few studies have addressed the prevalence and clinical importance of IOM lesions in elbow and forearm injuries. Two recent MRI studies on radial head injuries reported a surprisingly high prevalence of IOM lesions in the acute setting. In the first study, Hausmann et al. [18] assessed the presence of IOM injuries in 14 Mason type I radial head fractures; the authors found a 1–2 cm tear in the distal band of the interosseous membrane (dbIOM) in nine patients, a complete tear in one patient, and no lesions in four patients. Seven of the ten patients with confirmed dbIOM complained of pain localized in the distal third of the forearm. In the second study, McGinley et al. [19] evaluated the presence of a lesion of the cbIOM in 18 patients who had suffered a radial head fracture (13 Mason type I, 2 Mason type II, and 3 Mason Type III). The MRI examination revealed a lesion of the cbIOM in all patients with a Mason type II or III radial head fracture, while no injuries were observed in the 13 patients with a Mason type I radial head fracture. 

The results of these two MRI studies are of particular interest because they reported an unexpectedly high prevalence of IOM lesions in radial head fractures. It is reasonable to presume that in more complex injuries, such as CEI, the prevalence of the IOM lesions should be higher. This finding is even more noteworthy in view of the fact that the healing potential of the cbIOM is, according to the current expert opinion, poor [15,16]. Indeed, once the cbIOM has been injured, it is reported to lose its biomechanical properties and, consequently, the ability to maintain both the longitudinal and transverse stability of the forearm and to redistribute the mechanical loads between the radius and the ulna. It would therefore be reasonable to recommend a systematic assessment of the cbIOM in the acute setting and the treatment of its lesions to restore the proper function of the forearm and to avoid the chronic overload of the lateral compartment of the elbow, as recently suggested by some authors [12,13]. This point of view seems to also be supported by some clinical observations in patients affected by the sequelae of CEI. In particular, the early arthritic degeneration of the radio-capitellar joint and/or chronic lateral elbow pain, whose underlying cause was not clear, were observed in patients who had undergone the appropriate treatment for radial head fractures based on either a prosthesis or fixation [12,13,30,31]; in these symptomatic cases, a chronic overload of the lateral compartment of the elbow secondary to cbIOM chronic insufficiency was supposed to be the cause [12,13]. 

Currently, no studies have focused on the healing potential of the cbIOM as a means of confirming or rejecting the current expert opinion; furthermore, the prevalence of cbIOM insufficiency in the chronic CEI setting is as yet unknown, as is its clinical significance, considering that no studies have yet compared the prevalence of radio-capitellar arthritis and lateral elbow pain in patients with and without cbIOM chronic lesions. 

In view of these gaps in the currently available knowledge, we performed the present study to shed light on at least one of the issues regarding IOM lesions, i.e., we aimed to assess the prevalence and clinical significance of chronic cbIOM injuries after the appropriate treatment of Mason II and III radial head fractures in CEI. Our primary hypothesis, based on McGinley’s [19] results and current expert opinions, was that a high prevalence of chronic cbIOM was to be expected. A large sample of 93 patients affected by CEI with Mason type II or III radial head fractures were assessed using a bilateral US evaluation of the IOM. The bilateral US examination of the forearm was intended to detect any changes in the IOM characteristics (thickness, echoic aspect, continuity, etc.) with respect to the non-injured side, which are suggestive of a previous IOM injury.

We adopted US because it is more accessible and economical than MRI but is just as reliable [26,32,33,34,35,36,37]. In this regard, Fester et al. [33], in a cadaveric study evaluating the efficacy of MRI and US in detecting forearm IOM lesions, reported that the accuracy, the positive predictive value (PPV), the negative predictive value (NPV), the sensitivity, and the specificity of US and MRI were, respectively, 94%, 94%, 100%, 100%, and 89% and 96%, 100%, 93%, 93%, and 100%. Matsuoka et al. [26] assessed the accuracy, reliability, and usefulness of US to detect the normal or post-traumatic pathological aspects of the IOM in a bilateral examination of 46 forearms, including 36 from 18 healthy volunteers and 10 from 5 patients with limitation in forearm pronation/supination due to a previous injury. The US examination of patients with a previous forearm injury in the chronic setting revealed that, when injured, the interosseous membrane showed a frank discontinuity or hypoechoic traces when compared to the non-injured side. In the acute setting, the IOM lesions were identified as discontinuities of the membrane, and all of the preoperative US examination conclusions were confirmed intra-operatively. The ability of a US examination to detect chronic injuries in membranes, ligaments, tendons, and muscles was also reported by several other studies [34,35,36]. Interestingly, Christodoulou et al. [37] showed that the healed interosseous membrane of the leg had the same US aspect of an uninjured one when an appropriate fixation of both the tibia and fibula was performed in patients with ankle type B and C fractures according to the Danis–Weber classification [38]; they also reported a full concordance between the US examination results and the intra-operative findings.

The overall rate of cbIOM alteration in the present study was 5.4%, with no cases of positive “hernia signs” (i.e., complete rupture) and five cases with a slight increase in membrane laxity. None of the five patients displayed any clinical symptoms related to the chronic cbIOM lesion, although an asymptomatic non-union of the radial head associated with plate failure was observed in one patient. It should be noted that three of the five patients had a Monteggia-like fracture pattern, suggesting the higher prevalence of IOM involvement in such injury pattern [18,19,39,40,41]. 

The results of the present study are in contrast with what was previously reported in MRI studies [18,19], particularly when considering current expert opinions on the scarce healing potential of the cbIOM. In our study, the prevalence of cbIOM insufficiency was considerably lower than expected, which meant the null hypothesis of the present study was rejected. Such data seem to be supported by a lower incidence of IOM lesions in radial head fractures than previously reported. In the study by Awan et al. [42], no IOM tears were observed in the MRI forearm examination in an acute setting with 15 patients suffering from an isolated radial head fracture. In particular, the authors did not find any correlation between the severity of the radial head fracture according to the Mason classification and the extent of forearm soft tissue lesions (muscular and IOM edema with no discontinuity) or wrist pain observed. 

Another possible explanation for such contrasting results may be that the healing potential of the IOM is higher than previously believed, as hypothesized by Adams et al. [8,9] and Awan et al. [42]; Adams et al. [8,9] believed that the appropriate surgical treatment of CEI achieved by restoring the anatomy and the stability of the elbow–forearm–wrist functional unit allows the cbIOM to heal uneventfully. The importance of an appropriate surgical treatment in both acute and chronic CEI has also been previously highlighted by other studies [43,44]. Prospective studies focused on acute IOM lesions are needed to directly define their healing potential.

The secondary aim of the present study was to evaluate the clinical significance of chronic cbIOM insufficiency. However, the low prevalence of observed lesions did not allow us to draw any significant conclusion on this point. All but one cases with increased laxity showed satisfactory clinical and radiographic results; only one patient showed an asymptomatic radial head non-union potentially related to an impaired forearm load transmission mechanism. 

The possible limitations of the present study are due to (1) the lack of data on the prevalence of IOM lesions in the acute setting, which allowed us to draw only partial conclusions about the IOM healing potential, and (2) the small number of patients with chronic cbIOM lesions observed that prevented us from shedding light on their clinical significance.

Despite these limitations, we assessed in a large sample the prevalence of cbIOM chronic lesions in Mason II and III RH fractures associated with CEI, demonstrating their extreme rarity. Furthermore, this study highlighted the contrasting knowledge on this topic, pointing to the need for more clinical prudence when considering the ability of the IOM to heal, the role of chronic IOM lesions in lateral elbow pain, and the indications for the surgical reconstruction of this structure in the acute setting when the radial head is preserved or replaced.

## 5. Conclusions

Chronic lesions of the cbIOM in CEI with RH fractures are extremely rare. Considering the high prevalence of cbIOM lesions reported in the acute setting and the current belief about its scarce healing potential, the results of this study suggest that the frequency of such lesions may be lower than previously reported or that the healing potential of cbIOM lesions is considerably better than currently believed. Both of these scenarios warrant further dedicated studies to better understand the IOM’s pathophysiology and the clinical indications for its reconstruction.

## Figures and Tables

**Table 1:** Demographic data, injury pattern, surgical treatment, functional scores, and complications at last follow-up of patients with laxity at “hernia sign” test.

Patient	Age	Injury Patter	Surgical Treatment	Hernia Sign	ROM	Functional Score	Clinical Features	Radiological Signs and Complications
1, F	57	TTI: Mason II RH fracture; type I coronoid fracture *; MCL and LCL lesion; PL capsular lesion, lateral epicondylar muscle tendon lesion	RH arthroplasty; transosseous suture of coronoid; LCL and PL capsule repair with suture anchors; tendon suture	Negative but laxity	E/F 0/150° P/S 90°/90°	MEPS 95 MEPI excellent	Asymptomatic	Moderate radial neck SS; Grade I arthritis
2, F	76	Bilateral Monteggia-like fractures: Right side: olecranon fracture (Mayo type IIIA) type I coronoid fracture *; Mason III RH fracture with posterior dislocation (Bado II); LCL tear Left side: diaphyseal ulna fracture; Mason II marginal RH fracture with posterior dislocation (Bado II); LCL and PL capsular lesion	Right side: RH replacement; coronoid fixation with threaded wires; olecranon cerclage wire fixation; LCL repair with suture anchor Left side: ulna fixation with plate; RH fragment fracture removal; LCL and PL capsule repair with suture anchor	Negative but laxity Negative	E/F 10–150° P/S 85/85° E/F 5–150° P/S 85/85°	MEPS 95 MEPI excellent MEPS 95 MEPI excellent	Asymptomatic Asymptomatic	CH osteoporosis; moderate radial neck SS; Grade I arthritis Grade I arthritis
3, F	60	Monteggia-like fracture with diaphyseal fracture; type III coronoid fracture; Mason II RH neck fracture; posterior elbow dislocation	Ulna fixation with plate; coronoid fixation with 3 threaded K wires; RH plate fixation; LCL and PL capsule repair with two suture anchors	Negative but laxity	E/F 0–120° P/S 85/80°	MEPS 100 MEPI excellent	Asymptomatic	RH non-union with plate rupture
4, F	43	Elbow dislocation with Mason III RH fracture; MCL and LCL lesion	RH replacement; LCL repair with 5 mm suture anchor	Negative but laxity	E/F 40–120° P/S 85°/85°	MEPS 90 MEPI excellent	Asymptomatic	Moderate radial neck SS
5, M	51	Monteggia-like fracture; Mason II RH fracture with posterior dislocation; CH osteochondral fracture; LCL lesion	Ulna plate fixation; CH osteochondral fragment removal; RH fixation with headless screw; LCL suture anchors	Negative but laxity	E/F 0–145° P/S 80/80°	MEPS 95 MEPI excellent	Asymptomatic	Grade I lateral compartment arthritis

TTI: terrible triad injury; RH: radial head; PL: posterolateral; SS: stress shielding; LCL: lateral collateral ligament; MCL: medial collateral ligament; CH: capitulum humeri; E/F: extension/flexion; P/S: pronation/supination; MEPS: Mayo Elbow Performance Score; MEPI: Mayo Elbow Performance Index; * according to Regan-Morrey classification.

## Data Availability

The data presented in this study are available upon request from the corresponding author due to ethical and privacy reasons.
